# Perioperative Automated Noninvasive Blood Pressure- (NIBP-) Related Peripheral Nerve Injuries: An Anesthetist's Dilemma—A Case Report and Review of the Literature

**DOI:** 10.1155/2020/5653481

**Published:** 2020-06-29

**Authors:** Waleed Elmatite, Chanchal Mangla, Surjya Upadhyay, Joel Yarmush

**Affiliations:** ^1^New York-Presbyterian Brooklyn Methodist Hospital, New York, NY, USA; ^2^NMC Hospital DIP, Dubai, UAE

## Abstract

Peripheral nerve injury following regional or general anesthesia is a relatively uncommon entity but, potentially, a serious complication of anesthesia. Most nerve injuries are related to either regional anesthesia or position-related complications, and they are rarely seen in association with the use of automated blood pressure monitoring. We describe a patient who developed neurological dysfunction of all the three major nerves, median, ulnar, and radial, after general anesthesia. The distribution of sensory motor deficit along with the nerve conduction study demonstrated the location of the anatomical nerve lesions coinciding with the automatic noninvasive blood pressure (NIBP) cuff. No other cause of nerve injury was identified except for the use of the NIBP cuff. In the absence of another identifiable cause, we strongly suspected the NIBP cuff compression as a possible cause for the nerve injuries. In this article, we will discuss the possible risk factors, mechanisms, diagnosis, and prevention of perioperative nerve injury.

## 1. Background

Perioperative peripheral nerve injuries (PPNIs) are a serious perioperative complication that can be encountered both in general and regional anesthesia. Two ASA closed-claim analyses conducted a decade apart reveal that anesthesia-related nerve injuries are the third most common cause of anesthesia-related litigation claim [1]. Exact incidence of perioperative nerve injury is difficult to estimate because of the large variation in quality study design. Most of the data are from the retrospective analysis. Permanent peripheral nerve injury is extremely rare, and incidences of transient nerve injury ranges somewhere between 0.03 and 0.2 percent. [2, 3] Of the upper extremity, injuries to the ulnar nerve 28% and brachial plexus 20% are the more common perioperative nerve injuries, while injury to the radial (3%) and median nerve (4%) is less frequent. [1] In most cases with peripheral nerve injury, the exact mechanism of injury remains unclear [1]. We describe a rare case of acute perioperative nerve injury involving all three main nerves of the upper limb, radial, ulnar, and median nerves of the same arm. Prolonged use of the automated blood pressure cuff has been implicated in few case reports involving a single or two major nerves of the upper limb [4–7]. The incidence of automatic blood pressure cuff-related nerve injury is unknown, as it has been described only in case reports. All of the case reports showed single nerve injury, but in our case, all the major nerves of the left upper limb were affected.

## 2. Case Report

**Figure 1:**
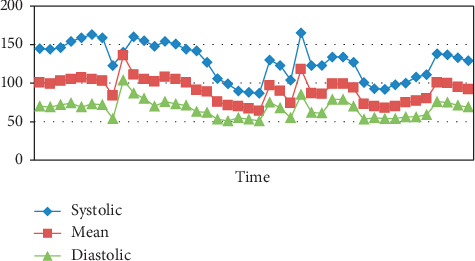
Trend of the blood pressure measurement.

A 64-year-old male with abdominal wall mass presented for excision of an anterior abdominal wall mass, adjacent tissue transfer, and flap graft under general anesthesia. His past medical history was significant for coronary artery disease status after three stents, hypertension, dyslipidemia, obstructive sleep apnea using home CPAP, and being a chronic smoker. His medication included clopidogrel (stopped 3 weeks before surgery), aspirin (stopped 5 days prior to the procedure), atorvastatin, hydrochlorothiazide-losartan, and metoprolol. He had no known allergies. His body mass index was 38 (height 183 cm and weight 129 kg). Intraoperative monitoring included continuous electrocardiograph (EKG), pulse oximetery, temperature, and automatic blood pressure monitoring with a large size adult blood pressure cuff (cuff size 16 cm width × 36 cm length and blood pressure monitor calibrated every 6–12 months) placed on his left upper arm, and possible pressure points were not found during a secondary survey. General anesthesia was induced through a 20 gauge intravenous catheter placed on the dorsum of the right hand. The patient was positioned supine with the head neutral and both arms placed neutrally alongside the body and secured on the arm board. Arm abduction was less than 90 degrees with padding of the pressure points. Intraoperatively, noninvasive blood pressure was measured every 5 minutes, and it ranged from 160 to 87 mm Hg systolic and from 104 to 51 mm Hg diastolic ( Figure 1 shows the trend of blood pressure measurements). Surgery lasted for about 9 hours (630 minutes). Throughout the surgery, the patient's temperature was maintained normothermic by using warm fluid, a forced air warming device, and maintaining the room temperature between 24°C and 26°C. At the end of the surgery, patient was extubated and transferred to the recovery room. In the recovery room, after two hours, the patient complained of left forearm swelling, pain, and wrist drop. Postoperatively, blood pressure was measured on the same arm every 10 min. Examination showed intact radial and ulnar pulse, variable sensory motor deficit-weakness of the dorsiflexion at the left wrist (grading 0/5), left wrist flexion (grading-3/5), inability to abduct the fingers and thumb opposition, weak left grasp 2/5, and normal power elsewhere including left shoulder abduction 5/5 left biceps and triceps 5/5, loss of sensation to light, touch, and pin prick to the left external forearm and hand, as well as a greater decrease in the sensation to left thumb, index, and middle fingers (median distribution) intact everywhere else (including the proximal left arm). Neurology consult confirmed the neurological findings. The impression was weakness and numbness distal to the left elbow affecting the left radial, median, and ulnar nerves consistent with compression neuropathies. Neurology consultation recommended physiotherapy and follow-up. Nerve conduction study after 3 weeks showed demyelination polyneuropathy. Nerve injury was confirmed by a conduction block distal to the spiral groove of the humerus, which was the level of upper extremity where the BP cuff was affixed, indicating that the injury was probably produced by the BP measurements. MRI brain did not show any abnormality. At the follow-up visit after 6 weeks, the patient was showing improvement in his clinical picture; he was able to perform opposition of the thumb, but still, he had residual weakness of the wrist dorsiflexion and all sensations were recovered.

### 2.1. Peripheral Nerve Structures

**Figure 2:**
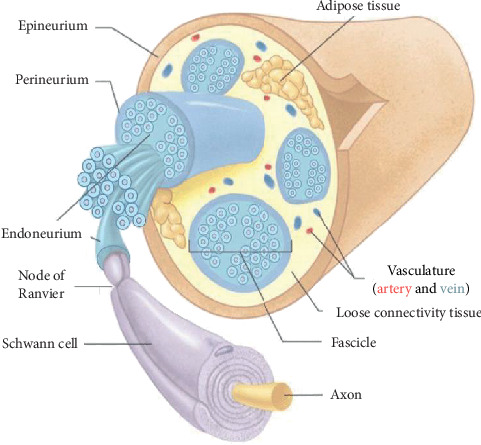
Peripheral nerve structures.

Peripheral nerves consist of the axons of neurons that reside in the central nervous system. Individual axons have a myelin sheath and Schwann cells that are surrounded by the endoneurium, which forms an unbroken tube around the axon from its origin in the spinal cord to the point where it synapses. A number of axons (nerve fibers) are organized into fascicles surrounded by a connective tissue layer, the perineurium. The number of fascicles increases, and their diameter decreases from proximal to distal with an increase in stromal tissue more distally. The epineurium itself is surrounded by the epineurium. The endoneurium acts as a selective barrier and produces endoneurial fluid (similar to the cerebrospinal fluid) that surrounds the axon. A peripheral nerve has an extrinsic plexus of vessels in the epineurial space that crosses the perineurium to anastomose with the intrinsic circulation of the endoneurium [8, 9] (Figure 2).

### 2.2. Pathophysiology of Peripheral Nerve Injury

Perioperative nerve injury can occur when a nerve is subjected to direct trauma, compression, stretch, hypoperfusion, or exposure to neurotoxic chemical or a combination of one or more factors. In most cases, the etiology remains unknown. The relationship between the pathophysiologic mechanism and the degree of neural injury is not straightforward. However, in the animal model of compression injury, the degree and duration of compression has been correlated with the degree of histological damage [8].

Patients with existing peripheral neuropathy (apparent or subclinincal), profound hypotension, hypoxemia, hypothermia, morbid obesity or underweight and malnourished, electrolyte imbalance, diabetes, current tobacco use, or anatomical variants are more susceptible to perioperative neural injury. A procedural risk factor such as the duration of surgery and patient position also increases the risk of neural damage [9]. Nerve injury is classified according to the Sedan and Sunderland classification, as shown in Table 1.

### 2.3. Mechanisms for Blood Pressure Cuff-Induced Nerve Injury

The exact mechanism of the peripheral nerve due blood pressure cuff injury is unclear, but the following are the possible mechanisms.

1. Direct mechanical compression differential pressure may be generated at the edge of the cuff may result in herniation of myelin sheath. This injury was confirmed by reversible deterioration of nerve conduction and even by electron microscopy. The main reasons for this mechanical compression are, first, the position of the pressure cuff close to the elbow where the radial, median, and ulnar nerves are more superficial and, second, the prolonged and frequent determination attempts to obtain blood pressure by the cuff due to excessive elbow movement. This type of injury is rarely seen in surgical procedures of the distal forearm with the use of a surgical tourniquet, although the arm is ischemic when using a pneumatic cuff because the elbow is immobile and there no mechanical force on the distal arm where peripheral nerves are superficial [12]. There are three important features of tourniquet paralysis that point to a mechanical origin of the injury: (a) fiber injury does not extend throughout the compressed area, but occurs only at the edge of the cuff; (b) small fibers are spared; and (c) the nodes of Ranvier are distorted and lost [12].
2. Nerve ischemia, 45 to 60 minutes of compression of 250 mmHg over the nerve, is required to reversibly block nerve conduction in the segment directly beneath the nerve. Even after three hours of compression at 250 mmHg, nerve conduction is slowed in the nerve segment distal to compression but not completely blocked. In our case, the maximum systolic pressure during surgery was 160 mmHg, making the seal off inflating pressure unlikely to approach or exceed 250 mmHg. The duration of inflation of the blood pressure cuff was around 100–150 seconds; accordingly, nerve ischemia may have less contribution than direct mechanical injury in pressure cuff-induced nerve injury [13].
3. Double crush syndrome, a compressible lesion occurring along a nerve, renders the nerve less tolerant of compression at the same or a second locus. Hence, nerves with a pre-existing injury or compression (e.g., patients with diabetes mellitus or patients suffering with rheumatoid arthritis with unstable joints) are at a greater risk of a second, possibly subclinical insult, resulting in a permanent nerve injury [14].
4. A combination of the abovementioned factors, as we suggest, is the reason for nerve injury in our case. Surgery was prolonged for 630 minutes. During the entire procedure, blood pressure measurements were performed noninvasively at least every 5 minutes with a single-sided blood pressure cuff. So, the most likely mechanism of nerve injury is direct nerve compression injury in addition to nerve ischemia caused by prolonged intermittent compression. Additionally, the patient has more risk factors including smoking, DLP, CAD, and HTN that predispose to peripheral atherosclerosis and nerve ischemia [15].

### 2.4. Risk Factors

Numerous factors have been observed to coincide with perioperative nerve injury, including surgical positioning during anesthesia, prolonged hypotension, hypothermia, prolonged application of a tourniquet (usually longer than 3 hours), the type of surgery (cardiac surgery), coexisting medical illness, hypertension, diabetes mellitus, pre-existing peripheral neuropathies, and smoking causing microvascular changes that may render these patients more susceptible to PPNI. The “double crush” syndrome through alteration in neuronal homeostasis may also play a role in this [15, 16].

### 2.5. Incidence

The incidence of nerve injury due to the blood pressure cuff is unknown. Eight cases of nerve injury related to the blood pressure cuff were published. Seven cases reported perioperatively (one case of ulnar nerve injury, one case of median nerve injury, and five cases of radial nerve injury) [4, 6, 17–20]. Mehta Reported one case of radial nerve injury related to ambulatory 24-hour blood pressure monitoring. The radial nerve injury is the most common nerve injury reported possibly due to its position over the lateral aspect of the humerus in the lower third of the arm, where the nerve courses from the posterior compartment to the anterior compartment of the arm immediately superior to the lateral epicondyle [19, 21]. So, it is more liable to direct mechanical compression. Yamada et al. reported a case of crush syndrome resulted from malfunction of the monitor resulting in continuous compression of the upper arm by an automatically cycled BP cuff. [22].Our case is different from the previously published cases as all the three major nerves of the upper limb were injured.  Table 2 shows the case reports of blood pressure cuff-induced nerve injury, the nerve injured, the type of surgery, the duration of surgery, and the possible risk factors.

### 2.6. Diagnosis

#### 2.6.1. Clinical Presentation

  Ulnar nerve injury (C7, C8–T1): it is usually characterized by a tingling sensation or numbness along the little finger and weakness of abduction, adduction of the fingers, or both. Examination of the hand reveals hyperextension of the themetacarpophalangeal joints and flexion at the distal and the proximal interphalangeal joints of the ring and the little finger (ulnar claw) [23].
  Radial nerve injury (C5–T1): wrist drop and numbness along the posterior surface of the lower part of the arm, posterior surface of the forearm, and a variable small area on the dorsum of the hand and the lateral three-and-a-half fingers [23].
  Median nerve injury (C5–T1): median nerve injury results in paraesthesia along the palmar aspect of the lateral three-and-a-half fingers. Motor manifestations include weakness of abduction and opposition of the thumb, weak wrist flexion, and the forearm being kept in supination. The muscles of the thenar eminence become wasted and the hand appears flattened [23].

#### 2.6.2. Electromyography (EMG) and Nerve Conduction Studies (NCSs)

  EMG: electromyography (EMG) involves recording the electrical activity of a muscle at rest and during volition from a needle electrode inserted within it. Abnormal spontaneous activity (fibrillation potentials; positive sharp waves due to denervated muscles) takes 1–4 weeks to develop and disappears with reinnervation. Their presence early on implies a pre-existing condition, and a specific etiological diagnosis cannot be made [23, 24].
  Nerve conduction studies (NCSs): NCSs evaluate the functional integrity of peripheral nerves and enable localization of the focal lesions. The compound sensory action potential will be reduced in sensory axonal degeneration if the electrodes overlie the affected portion of the nerve. In demyelination, there is focal slowing of sensory conduction or sensory and motor conduction across the injured portion of the nerve (proportional to the severity of the demyelination). NCSs are able to reveal the presence of a subclinical neuropathy predisposing nerves to injury and may also suggest the underlying pathological process (axon loss vs. demyelination), which has implications for the clinical course and prognosis [23, 24].
  NCSs and EMG are complementary and can help to determine whether a lesion is complete or incomplete; determine the basis of the clinical deficit; localize the lesion; define the severity and age of the lesion; and guide the prognosis and course of recovery [23, 24].

#### 2.6.3. Imaging Studies

  Three tesla magnetic resonance imaging provides adequate resolution to visualize peripheral nerves and may be used to identify and confirm the site of the lesion, particularly, if localization is undetermined by electrophysiological testing. High-resolution ultrasound has also been proposed as an adjunct to electrodiagnosis [23].

### 2.7. Recommendation of Authors to Avoid Pressure Cuff-Induced Nerve Injury

1. Locating the cuff higher on the arm, away from the elbow joints where *l* peripheral nerves are superficial to avoid direct mechanical pressure
2. Applying the proper size of pressure cuff according to the age and body weight to avoid excessive pressure when the cuff is so tight and to avoid repeated cycling of blood pressure monitoring due to a loose cuff
3. Decreasing the movement artifact at the elbow joint (by deep sedation for moving patients) to avoid and prolonged and repeated cuff cycle
4. Encourage using invasive blood pressure monitoring in prolonged surgery to avoid frequent cycling of the blood pressure cuff
5. For prolonged surgery, alternating the blood pressure cuff between two upper extremities may help to decrease the total duration of compression on the underlying nerves
6. Using soft cotton or woolen padding to protect the extremity may prevent this type of injury
7. Documentation of any previous preoperative peripheral nerve injury such as diabetic neuropathy, carpal tunnel syndrome, or cervical radiculopathy, as it is liable to double crush syndrome
8. Proper position during surgery to avoid peripheral injury and to avoid double crush syndrome.

## 3. Conclusions

Nerve injury caused by a blood pressure cuff is an uncommon event, but it can lead to significant morbidity of the affected patient. The exact mechanism of nerve injury is unknown, but may have resulted from mechanical compression at the lower edge of the pressure cuff on relatively superficially located peripheral nerves in the lower part of the arm. Prolonged surgery and repeated blood measurement are the most important risk factors. Locating the cuff higher on the arm, encouraging the use of invasive blood pressure monitoring, and alternating the blood pressure cuff between two upper extremities and in prolonged surgery may be considered to reduce the risk of peripheral nerve injury.

## Figures and Tables

**Table 1:** Classification of peripheral nerve injuries [10, 11].

Seddon	Sunderland	Pathophysiology
Neuropraxia (compression)	Type 1	Local myelin damage with the nerve still intact

Axonotmesis (crush)	Type 2	The continuity of axons is lost. The endoneurium, perineurium, and epineurium remain intact. Loss of continuity of axons with Wallerian degeneration due to disruption of axoplasmic flow
	Type 3 Type 4	Type 2 with endoneurial injury Type 2 with endoneurial and perineurial injury but an intact epineurium

Neurotmesis (transection)	Type 5	Complete physiological disruption of the entire nerve trunk. Early surgical intervention necessary. Prognosis guarded

**Table 2:** Case reports of blood pressure-induced nerve injuries [4, 6, 17–20].

Author	Nerve injury	Surgery	Duration of surgery	Risk factors
Swei et al. [20]	Radial nerve	Emergency appendectomy	120 minutes	Low body mass index (BMI) of 17.75, repeated inflation of the pressure cuff takes place in response to erroneous signals from outside interference

Bickler et al. [18]	Radial nerve	Lumbar epidural for normal labor	60 minutes	Actively moving patient during labor (moving artifact) leads to repeated prolonged measurement

Lin et al. [4]	Radial nerve	Laparotomy for perforated peptic ulcer	150 minutes	BMI 18.5, applying the pressure cuff at a lower level close to the cubital fossa

Van Ooijen et al. [6]	Ulnar nerve	Elective Le Fort iosteotomy.	240 minutes	BMI 20.6, prolonged surgery

Van Ooijen et al. [6]	Median nerve	Laparoscopic low anterior resection of the rectosigmoid colon	160 minutes	Hypertension, atrial fibrillation, and chronic obstructive pulmonary disease (COPD), prolonged and repeated blood pressure measurement

Kim et al. [5]	Radial nerves	Nail removal and nail lengthening for fibular hemimelia	180 minutes	Prolonged surgery

Mehta [19]	Radial nerve	Ambulatory 24-hour blood pressure monitoring	24 h	Hypertension, compression injury from 24-hour automated cycled blood pressure monitoring

Yamada et al. [22]	Unspecified nerve injury with the crush syndrome (rhabdomyolysis)	Total gastrectomy, splenectomy, and cholecystectomy for gastric cancer	340 minutes	Failure of deflation of the automatically cycled blood pressure, prolonged surgery
